# Author Correction: Global proteomic identifies multiple cancer-related signaling pathways altered by a gut pathobiont associated with colorectal cancer

**DOI:** 10.1038/s41598-023-44834-9

**Published:** 2023-10-17

**Authors:** Ewa Pasquereau-Kotula, Giulia Nigro, Florent Dingli, Damarys Loew, Patrick Poullet, Yi Xu, Scott Kopetz, Jennifer Davis, Lucie Peduto, Catherine Robbe-Masselot, Philippe Sansonetti, Patrick Trieu-Cuot, Shaynoor Dramsi

**Affiliations:** 1 Biology of Gram-Positive Pathogens Unit, Institut Pasteur, Université Paris Cité, CNRS UMR6047, 75015 Paris, France; 2 Stroma, Inflammation and Tissue Repair Unit, Institut Pasteur, Université Paris Cité, INSERM U1224, 75015 Paris, France; 3 Microenvironment and Immunity Unit, Institut Pasteur, Université Paris Cité, INSERM U1224, 75015 Paris, France; 4 Institut Curie, PSL Research University, CurieCoreTech Spectrométrie de Masse Protéomique, 75005 Paris, France; 5 Institut Curie, Bioinformatics Core Facility (CUBIC), INSERM U900, PSL Research University, Mines Paris Tech, 75005 Paris, France; 6 Center for Infectious and Inflammatory Diseases, Institute of Biosciences and Technology, Texas A&M Health Science Center, Houston, TX USA; 7 Department of Microbial Pathogenesis and Immunology, School of Medicine, Bryan, TX USA; 8 Department of Microbiology and Molecular Genetics, University of Texas Health Science Center, Houston, TX USA; 9 Department of Gastrointestinal Medical Oncology, The University of Texas MD Anderson Cancer Center, Houston, TX USA; 10 Université de Lille, CNRS, UMR8576-UGSF-Unité de Glycobiologie Structurale et Fonctionnelle, 59000 Lille, France; 11 Institut Pasteur, Unité de Pathogénie Microbienne Moléculaire, INSERM U1202, and College de France, 75005 Paris, France; 12 Present Address: University of Kansas, Kansas City, KS USA

Correction to: *Scientific Reports* 10.1038/s41598-023-41951-3, published online 11 September 2023

In the original version of this Article the authors’ given names and surnames were reversed.

The original Article has been corrected.

