# Supplementary figures and images for: Global proteomic identifies multiple cancer-related signaling pathways altered by a gut pathobiont associated with colorectal cancer
Source: Sci Rep. 2023 Sep 11;13:14960. doi: 10.1038/s41598-023-41951-3 (PMC10495336; doi:10.1038/s41598-023-41951-3)

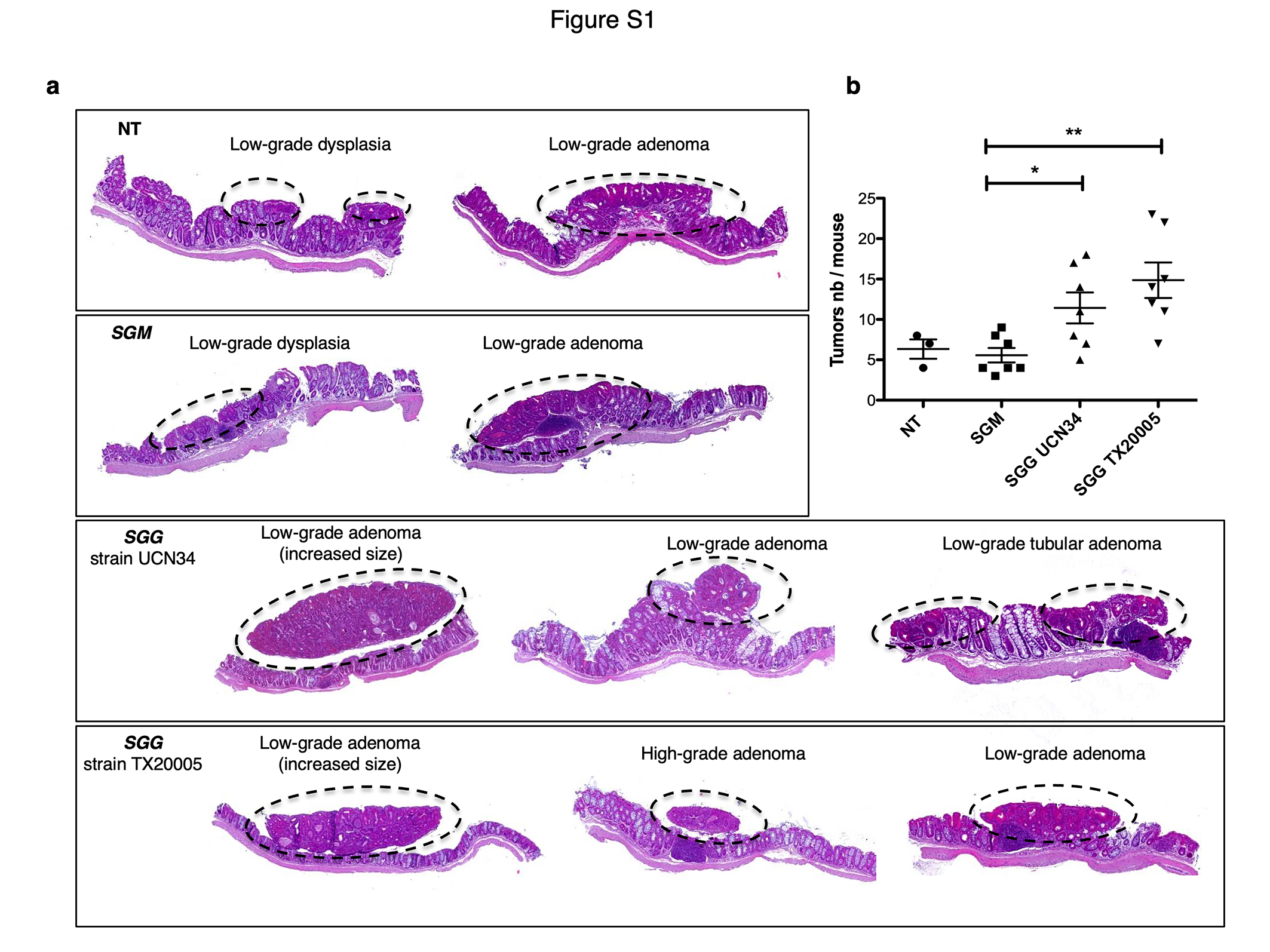

Supplement: Supplementary file 1 — Supplementary Figure 1. [file 41598_2023_41951_MOESM1_ESM.png]

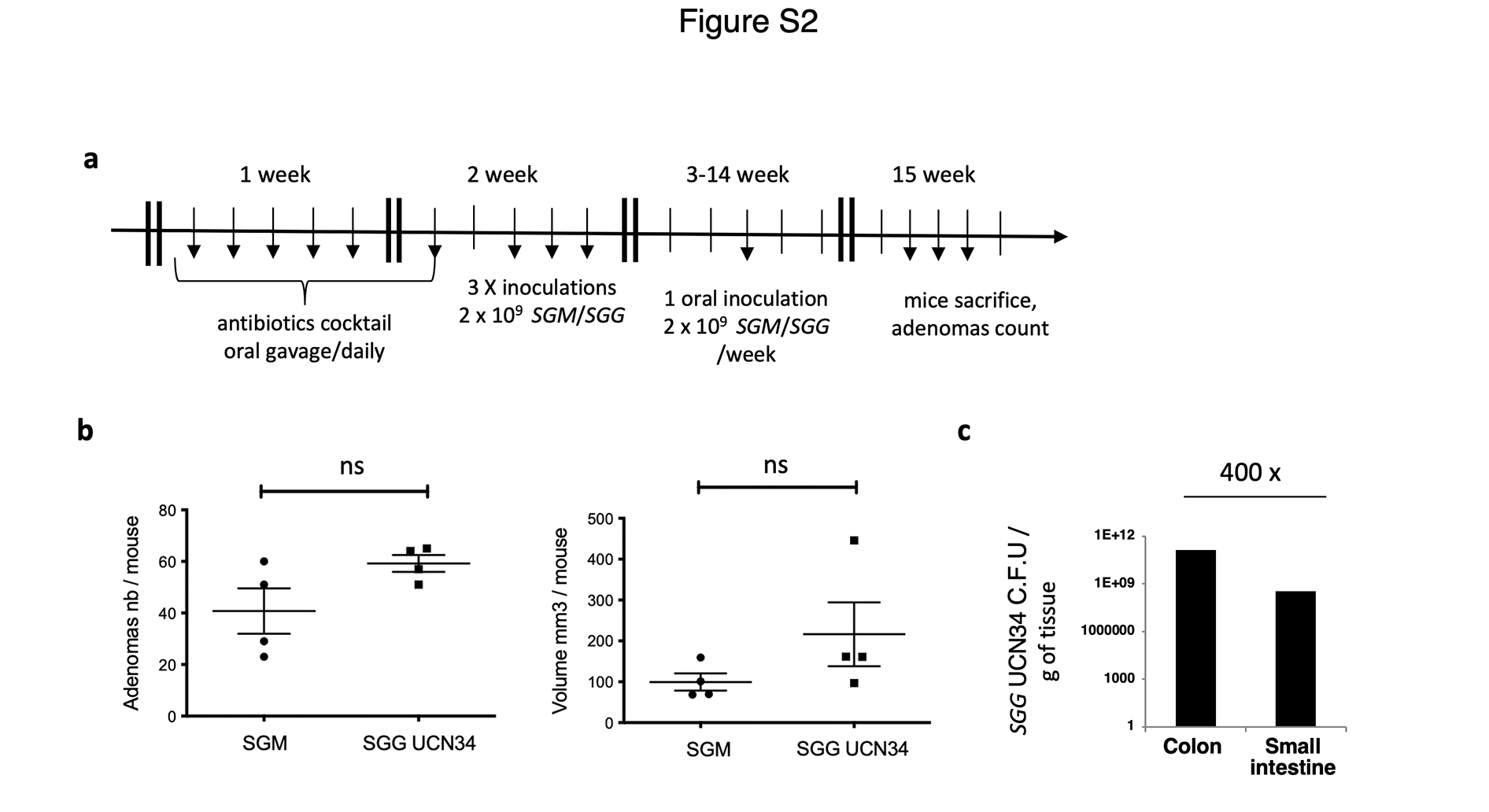

Supplement: Supplementary file 2 — Supplementary Figure 2. [file 41598_2023_41951_MOESM2_ESM.png]

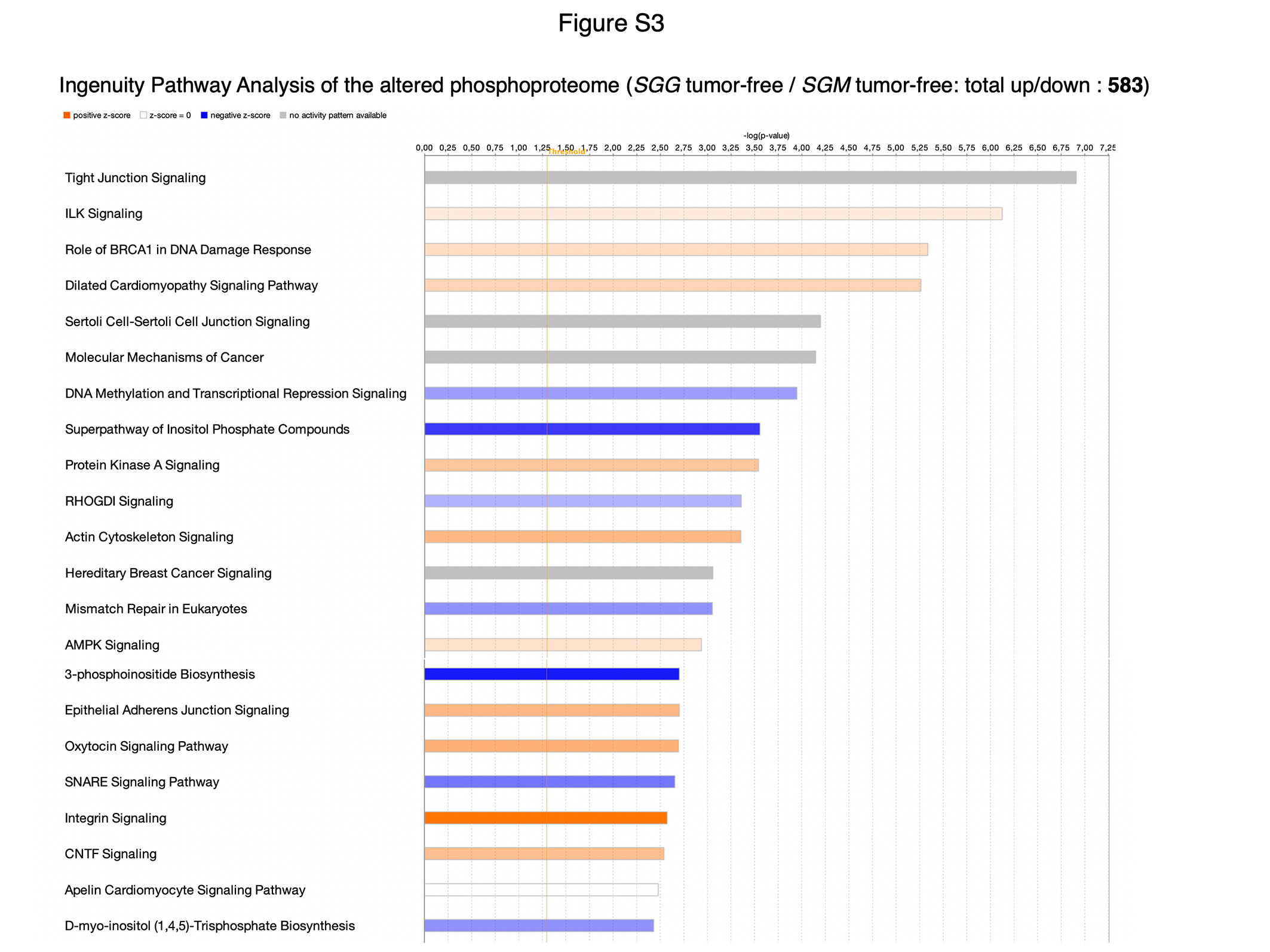

Supplement: Supplementary file 3 — Supplementary Figure 3. [file 41598_2023_41951_MOESM3_ESM.png]

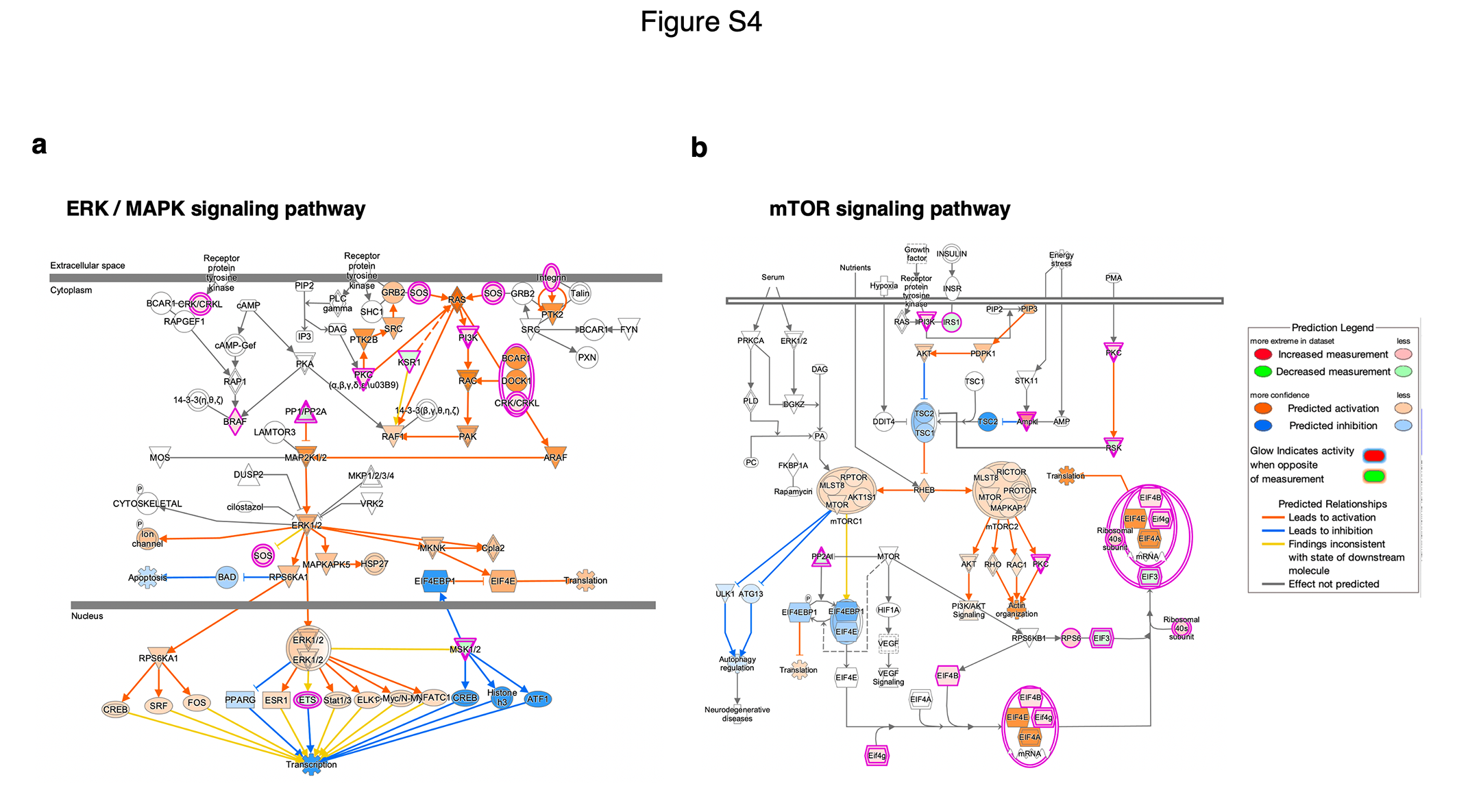

Supplement: Supplementary file 4 — Supplementary Figure 4. [file 41598_2023_41951_MOESM4_ESM.png]

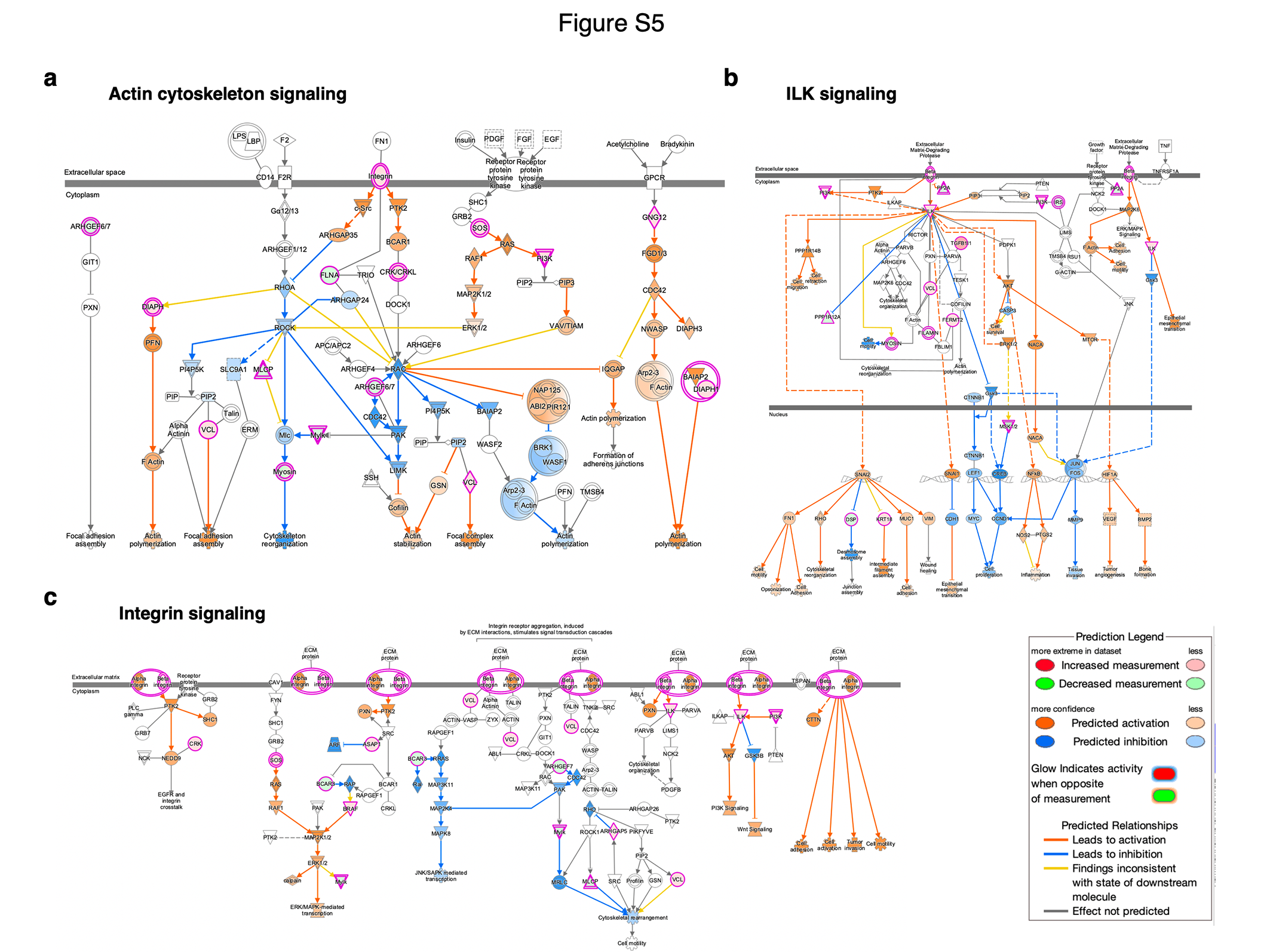

Supplement: Supplementary file 5 — Supplementary Figure 5. [file 41598_2023_41951_MOESM5_ESM.png]

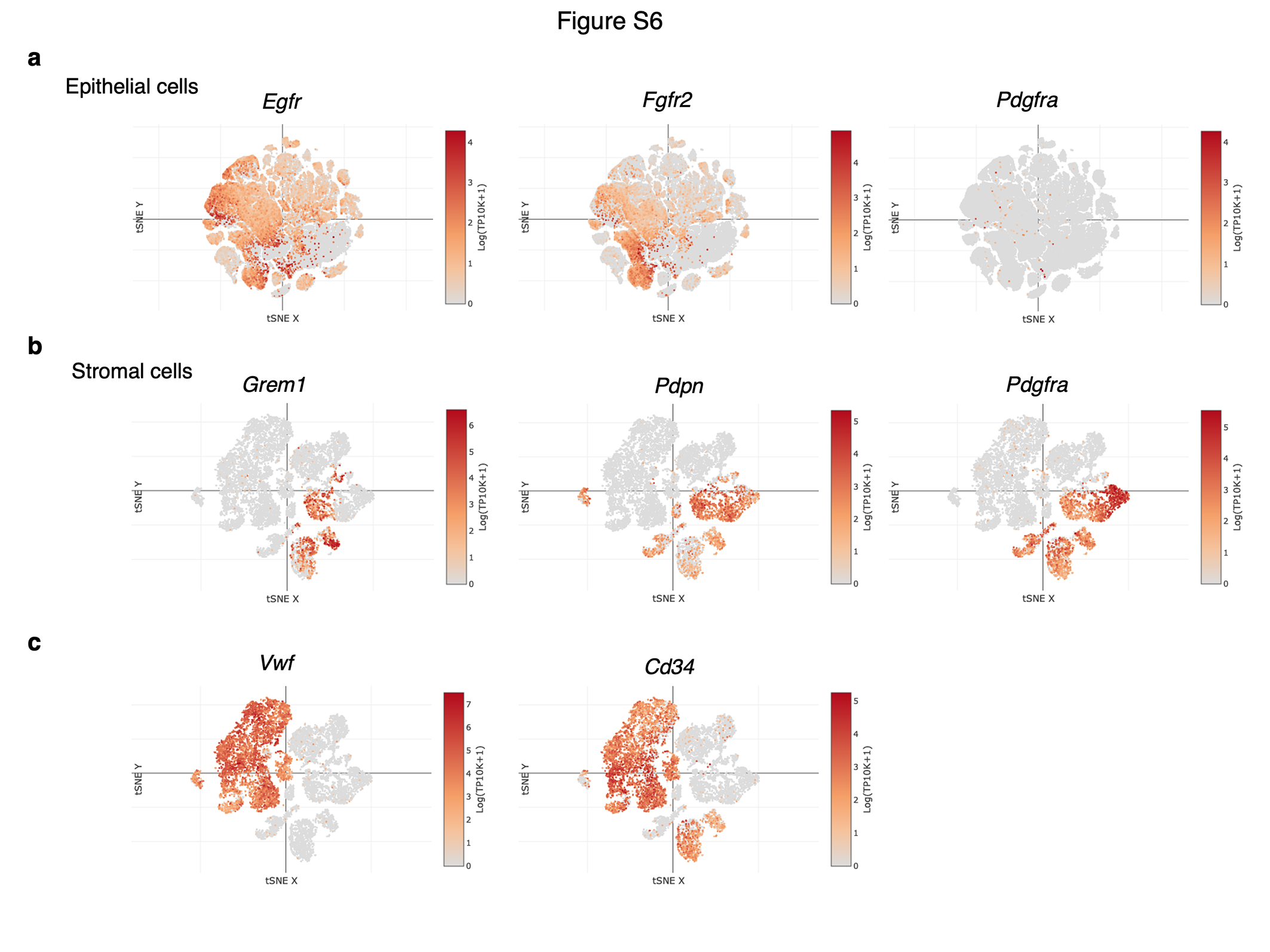

Supplement: Supplementary file 6 — Supplementary Figure 6. [file 41598_2023_41951_MOESM6_ESM.png]

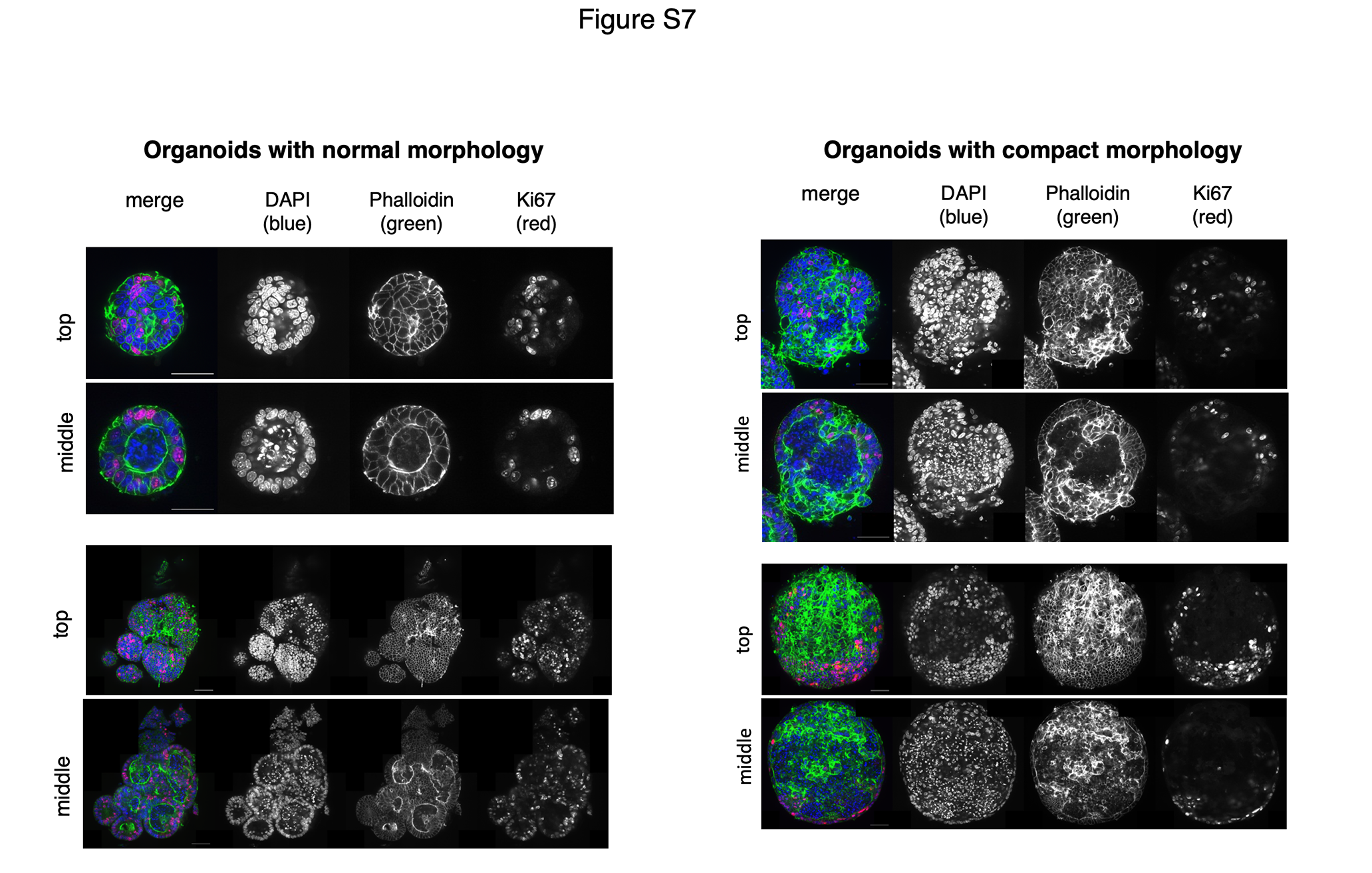

Supplement: Supplementary file 7 — Supplementary Figure 7. [file 41598_2023_41951_MOESM7_ESM.png]

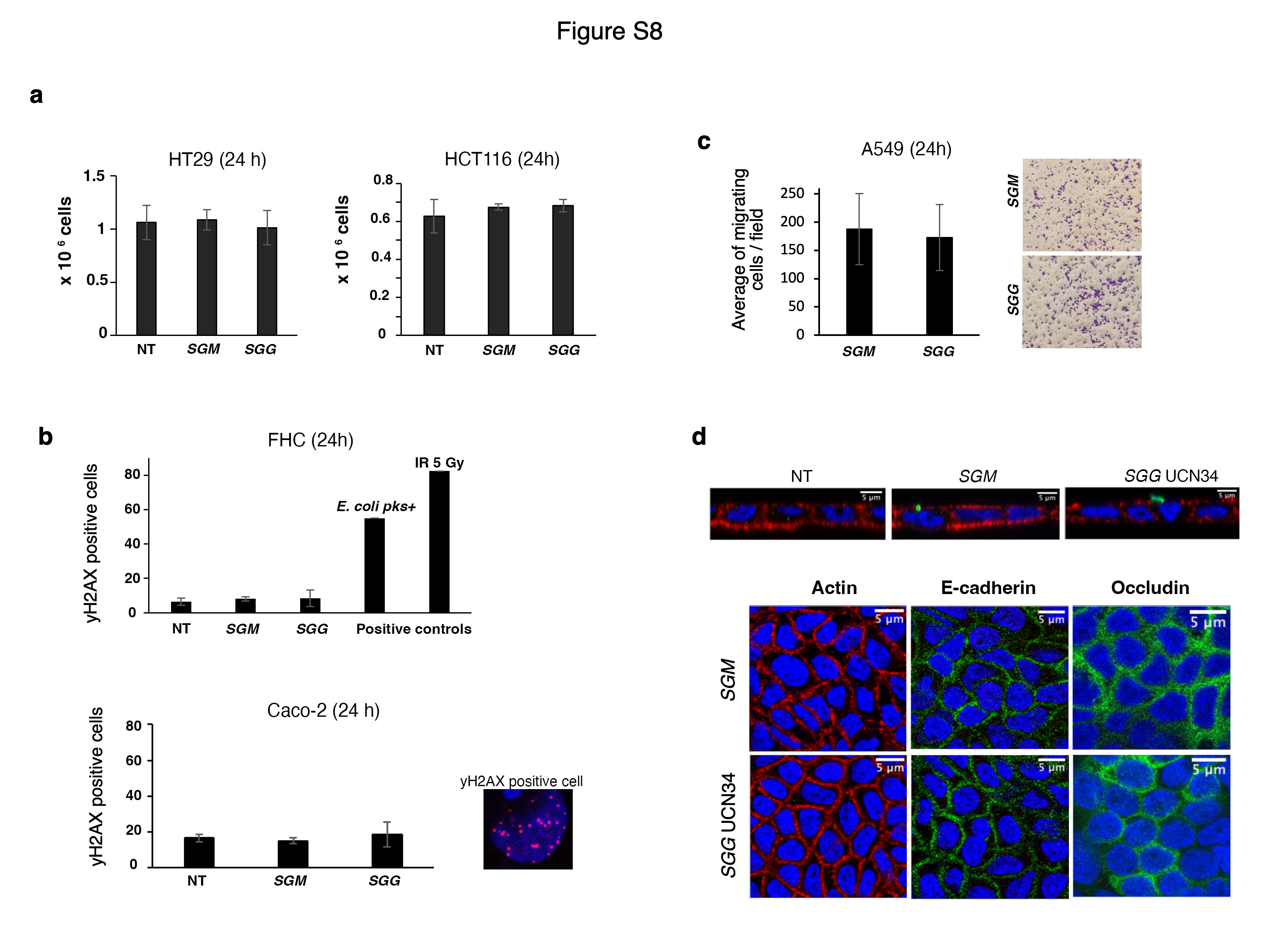

Supplement: Supplementary file 8 — Supplementary Figure 8. [file 41598_2023_41951_MOESM8_ESM.png]

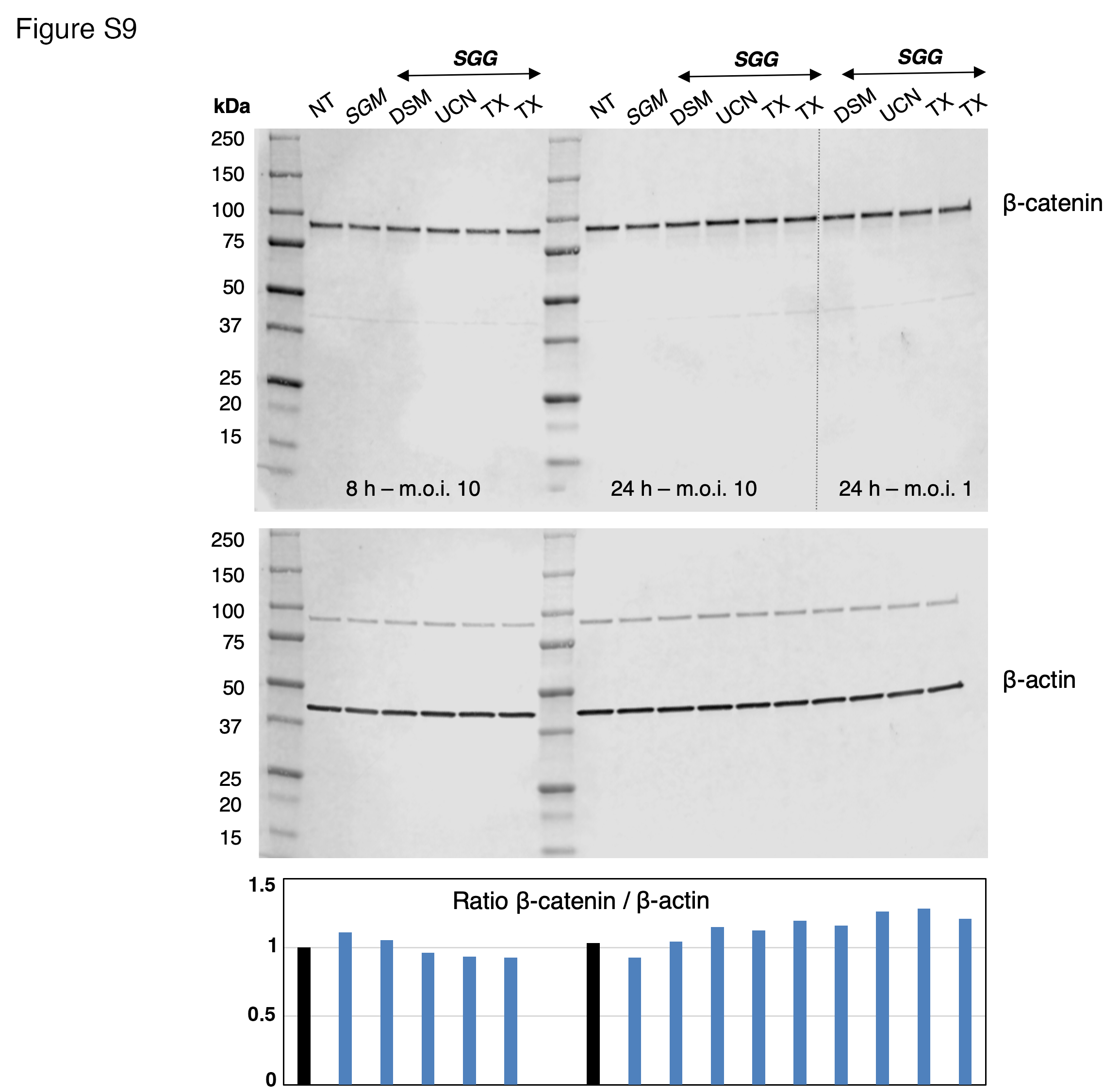

Supplement: Supplementary file 9 — Supplementary Figure 9. [file 41598_2023_41951_MOESM9_ESM.png]

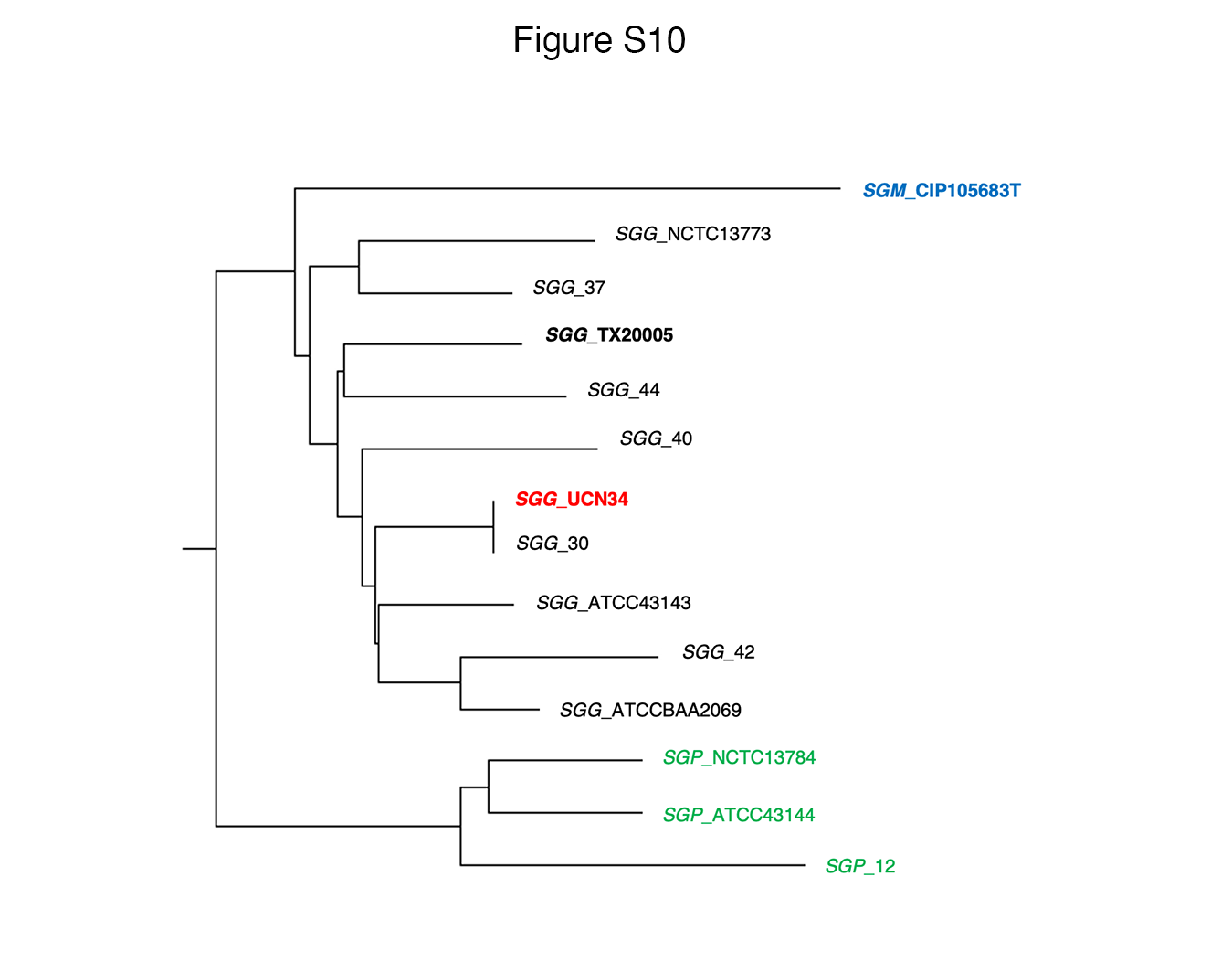

Supplement: Supplementary file 10 — Supplementary Figure 10. [file 41598_2023_41951_MOESM10_ESM.png]
